# A Mobile App for Thyroid Cancer Patients Aiming to Enhance Their Quality of Life: Protocol for a Quasiexperimental Interventional Pilot Study

**DOI:** 10.2196/13409

**Published:** 2020-03-13

**Authors:** Evanthia Giannoula, Ioannis Iakovou, Ioannis Katsikavelas, Panagiotis Antoniou, Vasilios Raftopoulos, Vasiliki Chatzipavlidou, Nikitas Papadopoulos, Panagiotis Bamidis

**Affiliations:** 1 Third Nuclear Medicine Department Papageorgiou Hospital Aristotle University of Thessaloniki Thessaloniki Greece; 2 Faculty of Engineering Aristotle University of Thessaloniki Thessaloniki Greece; 3 Medical Physics Laboratory Medical School Aristotle University of Thessaloniki Thessaloniki Greece; 4 Department of Nursing School of Health Sciences Cyprus University of Technology Limassol Cyprus; 5 Nuclear Medicine Department Theagenio Cancer Hospital Thessaloniki Greece

**Keywords:** mhealth, thyroid cancer, application, quality of life, patient-centered medicine, patient education

## Abstract

**Background:**

Thyroid cancer (TC) is one of the fastest growing cancers all over the world. Differentiated thyroid cancer (DTC) is the most frequent subtype of TC. When appropriate treatment is given, the prognosis for the patient is generally excellent. Despite the generally good prognosis of thyroid carcinomas, the symptoms may range from emotional to physical discomfort, depending on the thyroid hormone status, which can severely affect the patient. Moreover, the diagnostic and therapeutic procedures that DTC patients have to undergo, such as thyroidectomy and radioiodine therapy, significantly affect their mental and physical well-being. Often, the physician only addresses the favorable prognosis of DTC compared with other cancer types and neglects to assess issues related to the quality of life (QoL) of the patient; this was the reason we decided to design a mobile app for DTC patients and their caregivers.

**Objective:**

The aim of this study is to research the feasibility and applicability of an mHealth app tailored to DTC patients, as reflected in their QoL. The main features of the developed app offer access to useful information about thyroid cancer, diagnostic tests, and the appropriate therapy administered to DTC patients.

**Methods:**

Based on the existing literature, we created an up-to-date information platform regarding TC and especially DTC. In order to develop an effective app that can be implemented in current health care, we designed a section where the patient and physician can keep a medical record in an effort to enable access to such information at any time. Finally, we designed a user-friendly notification program, including pill prescription, follow-up tests, and doctor visit reminders in order to equally facilitate the lives of the patient and physician.

**Results:**

Having developed this mobile app, we aim to conduct a pilot quasiexperimental interventional trial. Our intention is to enroll at least 30 TC patients and assign them to intervention or control groups. Both groups will receive standard care for treating and monitoring TC, and the intervention group will also receive and use the DTC app. TC patients’ QoL will be assessed for both control and intervention groups in order to examine the effectiveness of the DTC app. QoL will be assessed through the QoL core questionnaire European Organisation for Research and Treatment of Cancer (EORTC) QLQ-THY34 in combination with the EORTC QLQ-C30 questionnaire through quantitative statistical analysis.

**Conclusions:**

The use of mHealth apps can play a significant role in patient education, disease self-management, remote monitoring of patients, and QoL improvement. However, the main limitation of the majority of existing studies has been the lack of assessing their usefulness as well as the absence of specific instruments to carry out this assessment. In light of those considerations, we developed a mobile app tailored to the needs of DTC patients. Furthermore, we evaluated its contribution to the QoL of the patients by using the EORTC QLQ-THY34 questionnaire, an accurate and safe instrument for the evaluation of the QoL in TC patients, while supporting future planned endeavors in the field.

**International Registered Report Identifier (IRRID):**

PRR1-10.2196/13409

## Introduction

Although thyroid cancer (TC) is a relatively rare neoplasm, accounting for approximately 1% to 5% of all cancers in females and less than 2% in males, it is the most common endocrine malignancy [1]. Furthermore, despite recent observations by Shi et al [2] that TC incidence rates for both sexes have declined since 2009, numerous other studies establish that thyroid gland neoplasms have shown the fastest increasing incidence of all malignancies over the past decades. This epidemiological trend, mostly attributed to overdiagnosis, has also been associated with several environmental factors [3]. According to the Surveillance, Epidemiology, and End Results Program of the National Cancer Institute, it is estimated that by the end of 2018 the number of new cases of TC will be 53,990 (3.1% of all cancers), and approximately 2060 patients will die of the disease [4]. Differentiated thyroid cancer (DTC) is the most frequent subtype of TC and includes papillary and follicular types and their variants [5]. When appropriate treatment is given, the prognosis of the disease is generally excellent. Taking into consideration that DTC epidemiological trends and favorable prognosis present a high 5-year survival rate (up to 98.2%) and an unimpaired life expectancy in most patients, it is crucial to ensure the quality of life (QoL) of the patient [6].

Surgery is the primary and most effective therapeutic approach for TC patients. Near total or total thyroidectomy with or without lymph node neck dissection is followed by radioiodine administration where appropriate. Thyroid stimulating hormone (TSH)-suppressive doses of thyroid hormone are administrated. This therapeutic management minimizes the risk of disease recurrence and metastatic spread while enabling accurate long-term surveillance. Systematic and targeted therapy is considered only for a small fragment of TC patients [7-10]. These therapeutic interventions, their preparation, possible complications or adverse events as well as the series of diagnostic and follow-up tests patients have to undergo may severely affect their QoL. TC patients often have difficulty in realizing the ways in which the disease will affect their everyday lives including, among other things, daily levothyroxine administration and frequent appointments with their doctors for diagnostic, therapeutic, and follow-up procedures. These procedures can be quiet stressful resulting in aggravating their mental health, especially in periods of hypothyroidism [11,12]. Moreover, the lack of proper information regarding the type of symptoms the patients will face, diagnostic and therapeutic procedures to which they are about to be submitted, and their impact on their physical and mental health accentuates feelings of isolation, fear, and anxiety [13,14]. Finally, caregivers play a crucial and demanding role regarding the care of TC patients, since they do not only exchange medical information with health care providers but also closely experience the disease of their loved ones to such an extent that they are often considered as a unit of care and may themselves experience mood disturbances and psychological impairment [15,16].

Proper patient education is of great importance as it ensures that the patient understands the potential side effects of their treatment. A patient who is informed and aware of all the side effects of the respective treatment is likely to be more tolerant of the treatment than an uniformed one [17]. It is also crucial for them to understand the seriousness of the potential side effects in order to comply with physician recommendations to help prevent or minimize radiation-induced sequelae. Educational programs are required to adequately prepare not only patients but also health care professionals for future care. In particular, recognition of the totality of the cancer experience and the need for both staff and patient education were illustrated by Stajduhar et al [18]. If health care professionals were providing comprehensive cancer care, psychosocial and physical needs would be equally addressed. Fulfilling these needs requires a collaborative approach among patients and health care professionals, and more modern approaches such as mobile health (mHealth) are also of great assistance [18].

The use of mobile phones and mHealth apps is constantly proliferating [19]. The increasing use of health apps has been documented among health care professionals, younger and higher educated patients, and the general public. Apps can play a significant role in patient education, disease self-management, remote monitoring of patients, improving patient and caregivers QoL [20]. There have been many papers and reviews on the topic of mHealth. Even in cases of chronic diseases, such as cancer, patients expressed their preference regarding the use of apps. In a recent editorial for a relevant special issue, Coughlin [21] outlined the proliferation of mHealth by exploring institutional guidelines for smartphone development in the United States and other countries [22,23]. Previously, Bender et al [24] found 1314 potentially relevant apps, out of which 309 met the selection criteria for their systematic review on smartphone apps for the prevention, detection, and management of cancer. Eleven apps provided tools to support the management of cancer. The majority of these apps (n=7) were not specific to a particular cancer type, and none of them was tailored for TC. These apps offered a combination of tools to assist the management of medical appointments, self-monitoring of symptoms, or medication consumption. The authors concluded that despite the existence of hundreds of cancer-focused apps, there is a lack of evidence regarding their utility, effectiveness, and safety [24]. Seiler et al [25] concluded, in a rather rigorous systematic review and meta-analysis, that very few mHealth apps targeted fatigued cancer survivors, however they revealed some rather interesting incidental results. First, most of the apps targeted healthy lifestyle maintenance, emphasizing dietary and physical activity goals; second, it was assessed that in most cases results were positive toward these goals [26-29]. Furthermore, in a systematic review/position paper, Nasi et al [30] succinctly outlined the mHealth environment. In short, they summarized the core feature that makes mHealth prolific, namely the flexibility of information availability in a wireless network. This was made apparent by the truism “a wireless network may be not mobile, but a mobile network must be wireless” [31]. Furthermore, this work managed to defined the key direction for mHealth apps which is not treatment itself but empowerment, information, and improvement in QoL for the patient [30]. In this study, it was also made clear that most of the literature in mHealth provided positive results and focused in chronic diseases and specifically cancer. More than a third of the surveyed literature (14/38, 37%) comprised cancer subtopics such as chronic cardiovascular, pulmonary, and metabolic diseases (diabetes, asthma, obesity). Given adequately established interest in the field, a 4-dimensional framework for mHealth performance was introduced: efficiency, effectiveness, clinical effectiveness, and QoL [30]. Based on this framework, the aforementioned literature, and the fact that TC is a disease that is radically treated within a relatively short treatment time, our focus in this study was the fourth dimension, QoL. In this context, we designed and developed the first iteration of DTC, an mHealth app dedicated to DTC patients; in this study we aim to examine its effectiveness in patient QoL. Since the main limitation of most research is the lack of validated instruments for the evaluation of mHealth apps utility and effectiveness, the European Organisation for Research and Treatment of Cancer (EORTC) QLQ-THY34 questionnaire was used in order to yield accurate conclusions regarding the effectiveness of our intervention.

## Methods

### Study Aims

The primary objective of our study is to evaluate the design and implementation of an mHealth app tailored to DTC. This app aims to assist patients undergoing treatment for the management of malignant thyroid neoplasms by educating and motivating both them and their caregivers, facilitating maintenance of their medical record, and organizing their medication and appointments with their doctors. In order to examine the effectiveness of our intervention, we intend to conduct a quasiexperimental study assessing impact of the intervention on patient QoL as recorded in the EORTC QLQ-THY34 and EORTC QLQ-C30 questionnaires to reach accurate conclusions regarding the effectiveness of our app.

### App Development

#### Content of the App

**Figure 1:**
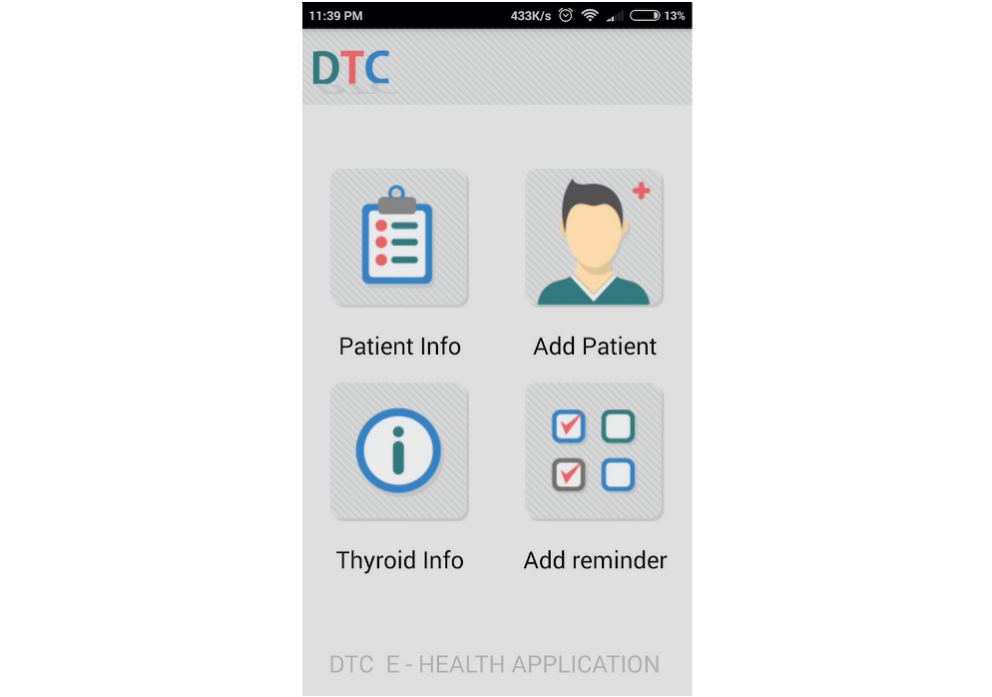
Screenshot of the DTC app home screen showing features Thyroid Info, Add Patient, Patient Info, and Add Reminder.

Our intention is to create an easy-to-use, accessible, and credible DTC app for patient QoL (Figure 1). We incorporated 4 different sections, with a specified function for each one of them. These included the core, informative Thyroid Info section which contains information regarding the thyroid gland and TC, especially DTC. The Add Patient and Patient Info sections enable both the doctor and patient to enter or view demographics and clinical data in order to keep an accessible medical record. Finally, the Add Reminder section includes an appointment organizer and pill reminder. The latter serves as an assistive tool to remind the patient to take daily levothyroxine and get properly prepared by undergoing thyroid hormone withdrawal or administering recombinant (rh)-TSH for diagnostic or therapeutic purposes.

**Figure 2:**
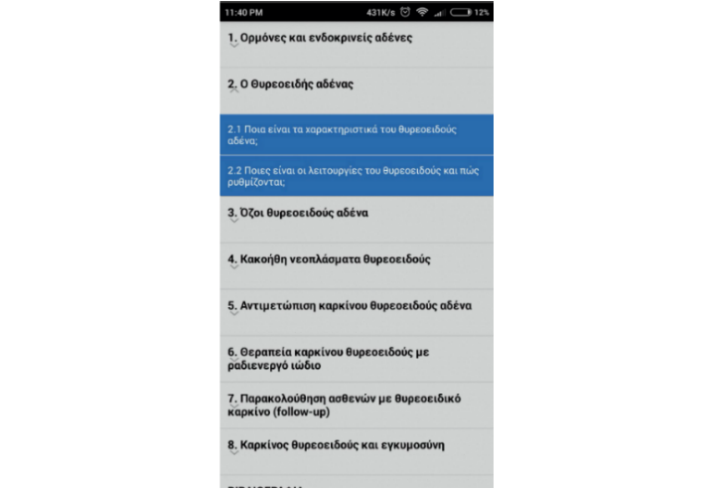
Thyroid Info feature comprises 8 chapters and references. Each chapter contains several questions so the patient can have the option to choose the topic they are interested in each time.

**Figure 3:**
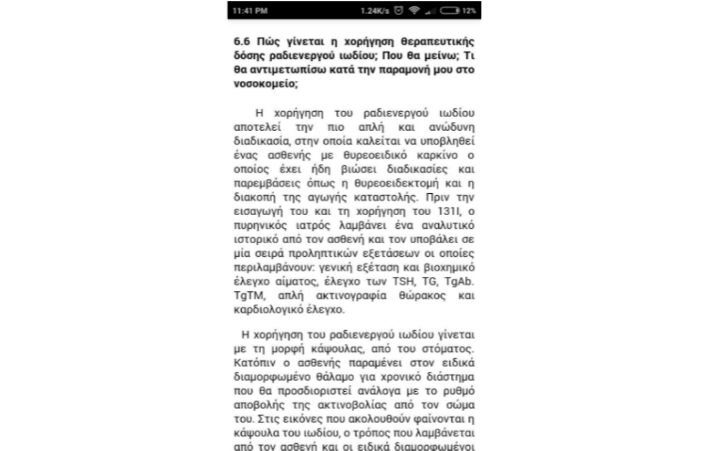
Screenshot of Thyroid Info feature content.

**Figure 4:**
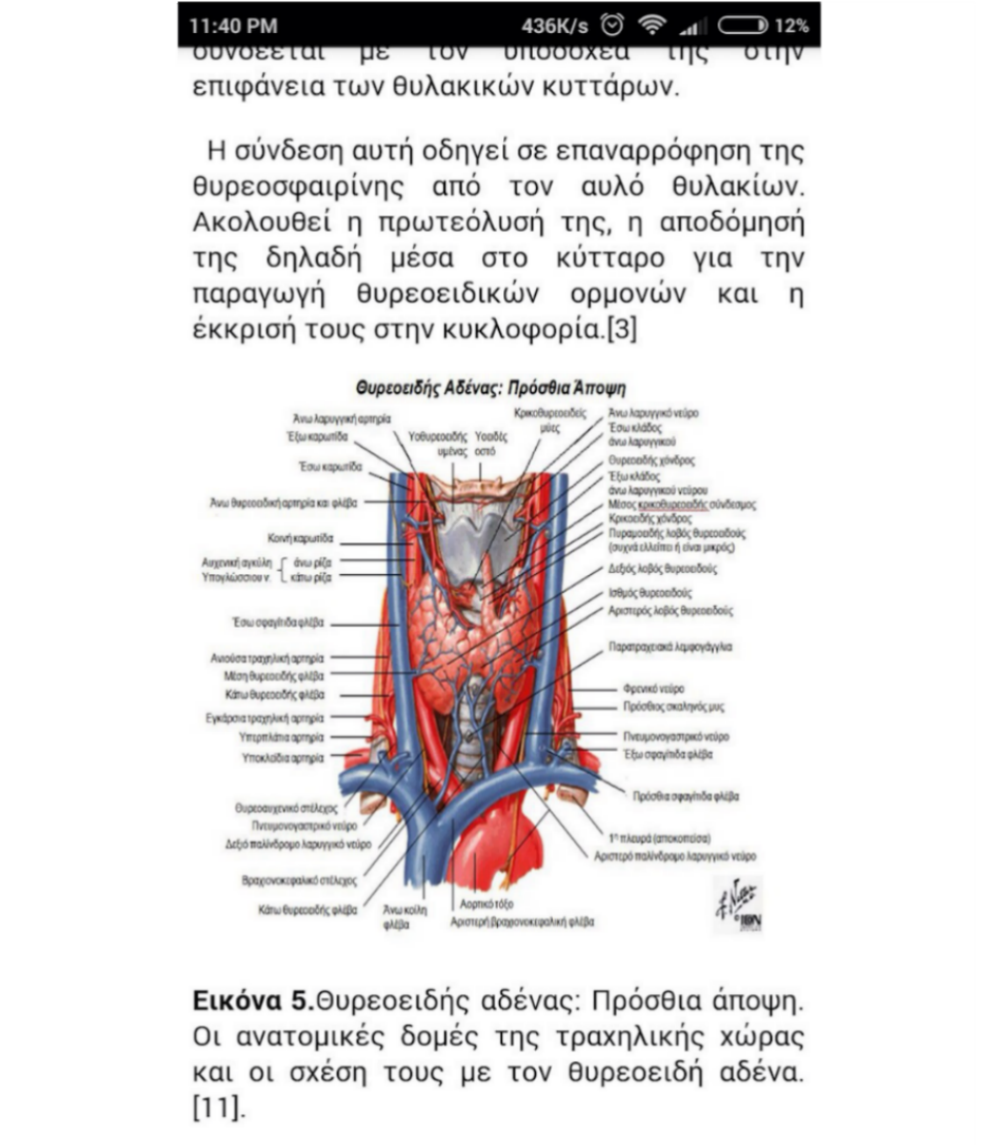
Screenshot of Thyroid Info feature content.

Τhe importance of proper information and education on TC patients’ effective disease management and therefore their prognosis, psychological status, and QoL is undisputed [17,18]. Given the amount of mostly nonvalidated and at times erroneous online information on thyroid malignancies, the majority of TC patients are rather misinformed and confused regarding their disease’s progress and the diagnostic and therapeutic procedures they must undergo. In this context, we intend to create and provide an educational tool for our patients, available any time through their mobile device (phones, tablets, etc). After reviewing the literature, focused mainly on guidelines for the management of TC, we developed an up-to-date, accessible, and easily understood information section, Thyroid Info, in clear and plain language (Figures 2 to 4). We conducted a literature search between September 2015 and July 2016 to review all aspects of thyroid function and neoplasms and their management from diagnosis to treatment and follow-up using the following electronic databases: PubMed, Highwire, and Google Scholar. The keywords and terms used in the literature research were: “hormones,” “thyroid gland,” “thyroid cancer,” “epidemiology,” “risk factors,” “management,” “diagnosis,” “treatment,” “radioiodine,” “adverse effects,” “guidelines,” “TC,” and “pregnancy.” These terms were used individually and in combination with the following links: or, and. Additional articles and reports were accessed via citations in reviewed papers that appeared in the original research. Our final database contains 71 publications. Our app is directed toward Greek patients, therefore we chose to use Greek in the app’s text. Thyroid Info consists of chapters on hormones and endocrine glands, thyroid gland, thyroid gland nodules, malignant neoplasms of the thyroid gland, management of TC, radioiodine treatment of DTC, follow-up of TC, TC and pregnancy, and the references we used. We structured Thyroid Info taking into consideration the respective literature and the disease issues that affect our patients. Each chapter is divided into several questions, allowing the patient to select the question that best matches their needs. Our text contains figures and tables rendering access to information easier and more targeted.

**Figure 5:**
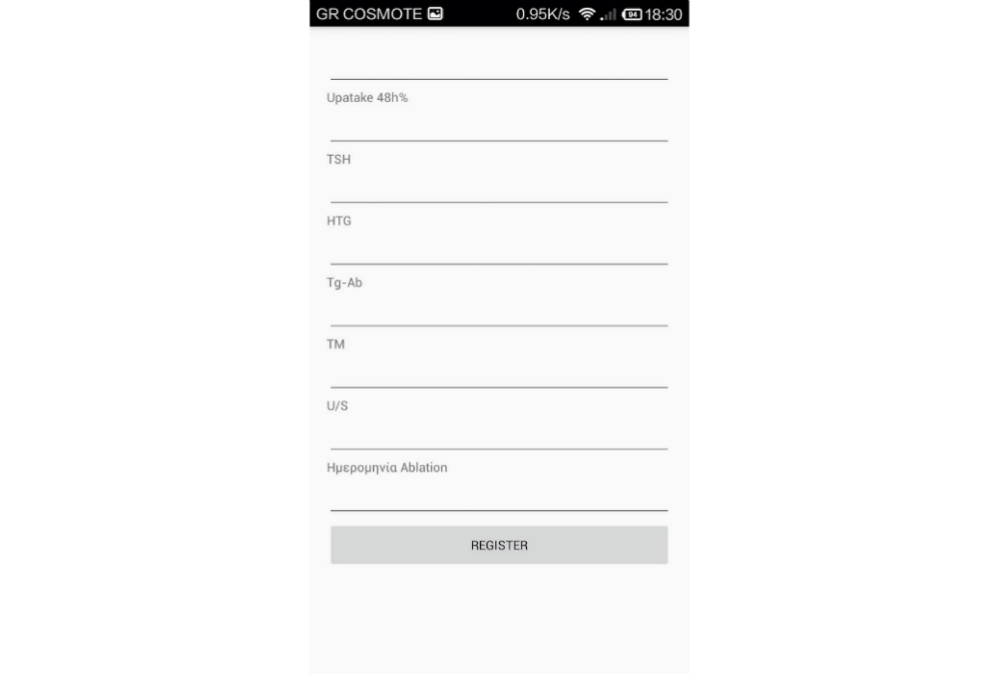
Screenshot of an Add Patient page. Registration can be done either in English or Greek.

In the Add Patient panel, the patient or physician can add relevant demographic and clinical information and the results of any follow-up tests (Figure 5). Information includes registration number, name, surname, username, password, email, code, phone number, address, gender, date of birth, age at diagnosis, date of surgery, name of surgeon, histology, maximum diameter of the largest TC foci, number of foci, thyroid gland lobe that TC was detected, invasion, number of excised lymph nodes, number of excised lymph nodes with metastases, stage, date of postsurgical remnant thyroid gland scintigraphy, date of radioiodine ablation, days between surgery and radioiodine ablation, preparation (thyroid hormone withdrawal or rh-TSH administration), I-131 scintigraphy, 24 hour uptake of I-131, neck-thyroid bed ultrasound, and levels of TSH, thyroglobulin, thyroglobulin antibodies, and thyroperoxidase antibodies. The Patient Info platform will serve as a medical record, containing patients’ registered data, available with the use of username and password set by the patient (Figure 5). The doctor or administrator has the option of adding new patients and their data from their personal computer.

The Add Reminder panel includes appointment organizer or reminder. This section was designed to serve as an appointment reminder and levothyroxine administration alert, enabling proper preparation for diagnostic and therapeutic procedures. The reminder will inform patient regarding when to withdraw thyroid hormone medication or rh-TSH injections in order to receive radioiodine for diagnostic or therapeutic purposes. Given that the volume of notifications received by users can be significant, we decided to use only a small number of notifications. When a patient visits the doctor, they can jointly arrange the next visit and prescribed medication and store this information in the app. In this context, we used Google’s Calendar API (application programming interface) to enable an organized display of the patient’s appointments and medication plans. The patient receives notifications for appointments a few days before the appointment and on the very day of the appointment. Regarding the medications, notifications are also used as a reminder. To best accommodate the needs of each patient, notifications can be edited.

#### App Design

The app was designed and developed in the Android Studio Development environment. The architecture used in this specific research study is a basic mobile architecture based on easy-to-use interface, system security, and data integrity. The app is easily deployable on any Android mobile platform, and plans are underway for the development of an iOS app. This app aims to provide a simple and intuitive interface, access to medical information (written by specialist doctors), and the ability to enable, store, and retrieve patient data (demographic and medical) in and from the cloud. The app also provides information to the user through notifications.

It is well known that the health care sector is extremely sensitive and has specific data protection requirements. Designing and developing a mobile app that contains sensitive patient data requires adherence to strict rules necessary to prevent unauthorized access to private information or personal data on the patient. It is also important to maintain updated log records for system failures. In view of the above, security has been one of our major concerns when designing and developing the DTC app. Data confidentiality and integrity has been ensured, while user and app authentication have been verified. This is the reason why we opted for cloud storage. Every data structure we store to the cloud is cryptographed. Tools such as hash functions, digital signatures, and symmetric and asymmetric keys are needed to achieve these features. Apart from that, we work on integrating the HL7 standards for the transfer of clinical and administrative data between software apps used by various health care providers.

Generally, the app design was geared to support the patient during treatment and management of DTC by informing and educating them. Furthermore, it was designed to empower them by keeping an up-to-date and easily accessible medical record while respecting their privacy, and providing reminders for medication and doctor appointments. Overall, the DTC mHealth app aims to become a key component in contributing to the improvement of patient QoL.

## Results

### Study Design

For this interventional study, we chose a quasiexperimental design to examine DTC patients’ QoL. The quasiexperimental approach was selected instead of the more rigorous randomized controlled trial (RCT) for two reasons. The first was the exploratory nature of this research since it is aiming to scope the features of such an app specifically pertaining to TC. The second is the necessity for credible but rapid results in order to iterate on the app itself. An RCT type of study is planned for a future iteration of the DTC app.

The main weaknesses of most of the previous studies regarding mHealth are the lack of assessment of the feasibility, usefulness, and effectiveness of mHealth apps and the lack of specific instruments for this assessment. As far as thyroid-related problems and functioning are concerned, only a few thyroid-specific QoL questionnaires have been developed. Moreover, we are informed assessments have been done largely using generic instruments. The EORTC created a TC-specific module to be used as a supplement to the QoL core questionnaire EORTC QLQ-C30 (phase I and II of the EORTC module development process) [32,33]. In these previous phases, items were derived from a longer list of QoL issues that could be relevant for TC patients. Phase III of the instrument development, according to the EORTC module development guidelines published in 2017, resulted in the EORTC QoL module for TC (EORTC QLQ-THY34) currently at the final phase IV validation which will be used in our study [34]. We acknowledge that using the not-yet-validated version of the EORTC THY34 is both a strong and a weak point of our study. By using the EORTC QLQ-THY34 questionnaire in combination with EORTC QLQ-C30 questionnaire, our intention is to reach safe and accurate conclusions given that both the app and questionnaire are tailored for TC [35].

### Participant Recruitment

Patients will be recruited at the 3rd nuclear medicine department of Papageorgiou Hospital, Aristotle University of Thessaloniki, Greece, and the nuclear medicine department of Theagenio Cancer Hospital, Thessaloniki, Greece. A formal protocol of the study has been submitted and approved by the ethics committee of the Aristotle University of Thessaloniki (no 398/11.12.2017) that shall include written consent from the patients to use their pseudonymized data in a statistically aggregate manner while respecting their privacy. We aim to enroll at least 30 patients into two groups without randomization. The first group will be given the DTC app, while the rest of the patients will receive oral instructions and informative printed material. Both groups will undergo standard diagnostic and therapeutic procedures the DTC patients receive. The inclusion criteria for patient participation in the intervention group are having undergone thyroidectomy, diagnosis of DTC, radioiodine treatment medically indicated, possession of mobile phone or tablet supporting Android software, and the ability to use Android apps. The control group will have similar demographic and clinical characteristics.

### Data Collection

For both groups there will be recorded demographic and clinical characteristics. As far as the intervention group is concerned, the data will be recorded by the nuclear medicine physician either via the DTC app on the patient’s mobile device or the physician’s personal computer. Following that, data will be exported in Excel (Microsoft Corp) files and stored in SPSS Statistics 25.0.0.0 (IBM Corp). Control group data will be recorded in Excel files and then stored in SPSS Statistics 25.0.0.0. Patients of both groups will be given the EORTC QLQ-THY34 and EORTC QLQ-C30 questionnaires to examine their QoL. This is the only instrument in the native language of the patients, developed by the EORTC QoL Group. The EORTC QoL module for TC consists of 34 items and is currently being pilot-tested for the final international field validation in phase IV. It will be available in the following languages: Arabic, Chinese, Dutch, English, French, German, Greek, Hebrew, Hindi, Japanese, Italian, Polish, Portuguese, and Tamil [32]. The module has been developed according to the EORTC guidelines and approved after formal review. The EORTC QLQ-THY34 is still under development and is thus being shared only with those groups willing to provide data relevant to evaluating its psychometric properties. In this framework, we received authorization to use the EORTC QLQ-THY34 questionnaire, assuring our compliance with the EORTC’s rules. Considering that the EORTC QLQ-THY34 questionnaire is formally translated and validated in Greek, it is appropriate to argue that it constitutes the most accurate and safe instrument to evaluate QoL in TC patients, thus ensuring the validity of our results on the effectiveness of the DTC app.

### Data Analysis

All data will be downloaded and stored in SPSS Statistics. Descriptive statistics will be used to characterize the overall sample, each condition, and study acceptability and demand. To examine preliminary differences in feasibility, we will use chi-square tests, Fisher exact tests, and nonparametric Mann-Whitney *U* tests for nonnormal data at the level of significance of *P*<.05 as appropriate.

Clinical evolution questions and diagnostic markers shall be checked for possible psychological effects of better information (eg, question 34: Have you experienced any throat ache?). However, the respective analysis shall focus on questions describing both the emotional disposition of the patient (eg, Did you feel distressed or irritated?) and their perceptions about the quality of care provided (eg, Did you feel well supported by your doctors?). The hypothesized scales assessed for our patients through the questionnaires are anxiety, body image, cramps, discomfort in the head and neck, dry mouth, fatigue, hair, impact on job or education, joint paint, neuropathological problems, restlessness, shoulder functioning, social support, swallowing, temperature tolerance, voice, and worries about important others.

## Discussion

### Mobile Health in the Service of Thyroid Cancer Patients

TC is a malignant neoplasm with excellent prognosis in cases where appropriate treatment is given. The standard therapeutic interventions, their preparation, possible complications or adverse events, and numerous diagnostic and follow-up tests that TC patients must undergo may severely affect their QoL. Evidence-based medicine, a more personalized patient approach, novel management strategies such as the use of rh-TSH, and proper patient education are intended to ensure QoL. The use of mHealth apps—currently proliferating—can play a significant role in patient education, disease self-management, and remote monitoring of patients, thus resulting in an improvement in their QoL. There have been already several papers and reviews on the topic of mHealth addressed in conjunction with chronic diseases and, more specifically, with cancer. However, the key weakness of most of the studies to date has been the lack of assessing their usefulness and specific instruments used in the assessment. In this context, we attempted to develop a mobile app tailored to DTC patients and evaluate its contribution to patient QoL by using the EORTC QLQ-THY34 questionnaire. To the best of our knowledge, the QLQ-THY34 questionnaire is the most accurate and safe instrument to evaluate QoL in TC patients and is also under validation and translated in Greek, which ensures the validity of our results on the effectiveness of the DTC app.

The work described here is a precursor to a planned RCT endeavor on the impact of such solutions on the overall QoL aspect of cancer survivorship, an active and open research endeavor. This work aims to assess how effective the app is regarding the improvement of QoL of these patients (soon to be considered as productive and healthy individuals), evaluate the DTC mHealth solution regarding the patient’s access to medical information, and provide moral support during and after treatment. This shall be achieved with validated evaluation instruments through accepted common practices at two of the largest hospitals in Greece. This quasiexperimental study shall provide evidence-based feedback for the iterative refinement of this mHealth solution and support future planned endeavors and research in the field.

## Abbreviations

API: application programming interface
DTC: differentiated thyroid cancer
EORTC: European Organisation for Research and Treatment of Cancer
mHealth: mobile health
QoL: quality of life
RCT: randomized controlled trial
rh-TSH: recombinant thyroid stimulating hormone
TC: thyroid cancer
TSH: thyroid stimulating hormone
